# A novel adhesive factor contributing to the virulence of *Vibrio parahaemolyticus*

**DOI:** 10.1038/srep14449

**Published:** 2015-09-24

**Authors:** Ming Liu, Sheng Chen

**Affiliations:** 1 Shenzhen Key Lab for Food Biological Safety Control, Food Safety and Technology Research Center, Hong Kong PolyU Shenzhen Research Institute, Shenzhen, China; 2 State Key Laboratory of Chirosciences, Department of Applied Biology and Chemical Technology, The Hong Kong Polytechnic University, Hong Kong, China

## Abstract

Bacterial adhesins play a pivotal role in the tight bacteria-host cells attachment to initiate the downstream processes and bacterial infection of hosts. In this study, we identified a novel adhesin, VpadF in *V. parahaemolyticus*. Deletion of *VpadF* in *V. parahaemolyticus* markedly impaired its attachment and cytotoxicity to epithelial cells, as well as attenuated the virulence in murine model. Biochemical studies revealed that VpadF recognized both fibronectin and fibrinogen. The binding of VpadF to these two host receptors was mainly dependent on the its fifth bacterial immunoglobulin-like group domain and its C-terminal tail. Our finding suggested that VpadF is a major virulence factor of *V. parahaemolyticus* and a potential good candidate for *V. parahaemolyticus* infection control for both vaccine development and drug target.

*Vibrio parahaemolyticus* is an inhabitant of estuarine, marine and coastal environments. It is commonly free swimming and attaching to aquatic animals, including corals, fish, oyster, sponges, shrimp and zooplankton^1^,^2^. *V. parahaemolyticus* has also been recognized as the causative agent of seafood-related gastroenteritis, wound infections and septicemia^3^.

To survive in these diverse and challenging environments, *V. parahaemolyticus* is presumed to evolve rapidly. Molecular profiling studies on environmental and clinical isolates, as well as comparative genomic analysis of pre-pandemic and pandemic strains, reveals that *V. parahaemolyticus* genomes are highly versatile, and the emergence of pandemic strain could be associated with recombination and novel gene acquisition^4^,^5^,^6^,^7^. For instance, thermostable direct hemolysin (*tdh*), *tdh* related hemolysin (*trh*) and type III secretion system 2 (T3SS2) are commonly found within the pathogenicity island (Vp-PAI) from clinical isolates, suggesting that *V. parahaemolyticus* strains acquire these virulence determinants by horizontal gene transfer (HGT)^8^. TDH and TRH directly lead to cytotoxicity and enterotoxicity while T3SS2 is essential for the enteritis, colonization and competition to protists in aquatic environment^9^,^10^,^11^,^12^. In addition to TDH, TRH and T3SS2, some *V. parahaemolyticus* strains also acquire other virulence factors, such as VpaH and ZnuA^13^,^14^. These findings reinforce the notion that *V. parahaemolyticus* is a versatile pathogen with its virulence tightly linked to the acquisition of virulence factors through HGT.

The attachment of pathogens to host cells is the prerequisite for the efficient translocation of effectors that suppress host immune response and/or modulate cellular signaling pathways to aid its infection. Moreover, adhesion subverts host actin cytoskeleton and triggers cellular signaling pathways to recruit downstream signaling proteins to the plasma membrane to facilitate subsequent pathogen invasion. Attachment also ensures pathogens persistence in host niches^15^,^16^,^17^. To date, the multivalent adhesion molecule 7 (MAM7), mannose-sensitive haemagglutinin (MSHA) pilus, enolase, capsular polysaccharide and two type VI secretion systems (T6SSs) have been reported to contribute to cell attachment of *V. parahaemolyticus*^18^,^19^,^20^,^21^,^22^. However, among these adhesive organelles, only *mam7* was found to be required for the pathogenesis of *V. parahaemolyticus* in the worm infection model. MAM7 is a transmembrane protein that consists of a transmembrane motif at the N-terminus and seven mammalian cell entry domains that mediate its adherence to different types of cells by binding to fibronectin (Fn) and phosphatidic acid in the initial stage of infection and subsequent cytotoxicity against 3T3 fibroblasts and RAW264.7 macrophages, but not to HeLa or Caco-2 epithelial cells. Sequence homology search showed that MAM7 is not only present in *V. parahaemolyticus*, but also a conservative protein possessed by several Gram-negative pathogens, including enteropathogenic *Escherichia coli*, *Vibrio cholerae* and *Yersinia pseudotuberculosis*^22^. Given that each Gram-negative pathogen usually produces more than one adhesive factor to initiate infection^23^,^24^,^25^ and *V. parahaemolyticus* frequently acquires virulence factors, it is reasonable to speculate that *V. parahaemolyticus* has evolved or acquired specific, yet poorly understood mechanism to strengthen host-pathogen interactions. In this study, we identified a novel adhesin gene *vp1767*, which was referred to as *VpadF* (*V. parahaemolyticus* adhesive Factor), contributing to the attachment to and cytotoxicity against epithelial cells. This gene is essential for the lethal effect of *V. parahaemolyticus* on mice. Most importantly, VpadF bound to cell surface receptors, fibronectin (Fn) and fibrinogen (Fg), which may contribute to its host colonization and pathogenesis.

## Methods

### Bacterial strains, plasmids and growth conditions

*V. parahaemolyticus* strains, *E. coli* strains and plasmids used in this study were listed in **ST 1**. *V. parahaemolyticus* strains were cultured in LB medium supplemented with 2.5% sodium chloride (LBS) at 37 °C. Thiosulfate-citrate-bile salts-sucrose agar (TCBS) was used to select *V. parahaemolyticus* strains.

### Construction of gene deletion and complementary strains

The *vp1767* gene was deleted from clinical *V. parahaemolyticus* strain VP3218 by homologous recombination as described previously^14^. Similar procedures were used to obtain Δ*vcrD1* and Δ*vcrD1*Δ*VpadF* using specific primers (**ST 2**). To construct *VpadF* complementary strain, DNA fragment encoding *vp1767* gene with a C-terminal Flag tag and N-terminal ribosome binding site was amplified using primers vp1767com-F and vp1767com-R (**ST 2**). PCR product was digested and cloned into the same digested pMMB207 to create pMMB207:*VpadF*. The recombinant plasmid was transformed to *E. coli* SY327 *λpir* and then conjugated to *V. parahaemolyticus* strains with a helper partner *E. coli* SY327 *λpir* carrying pPK2013. Transconjugants were selected on TCBS containing 5 μg/ml chloramphenicol to obtain the complementary strains.

### Bioinformatics analysis

Protein domain search was performed using PFAM (http://pfam.sanger.ac.uk/search), InterPro (http://www.ebi.ac.uk/interpro) and SMART (http://smart.embl-heidelberg.de). The subcellular location and the transmembrane region were predicated using TMHMM Server v2.0 (http://www.cbs.dtu.dk/services/TMHMM/) and TMpred (http://www.ch.embnet.org/software/TMPRED_form.html). Multiple sequence alignments were achieved using Clustal W2 (http://www.ebi.ac.uk/Tools/msa/clustalw2/). Phylogenetic analysis was performed using MEGA version 5 after multiple alignment of the data via CLUSTAL_X. Distances were obtained using options according to Kimura’s two-parameter model and clustering was performed by using the neighbor-joining method. The topology of the neighbor-joining phylogenetic tree was evaluated by using bootstrap resampling with 1000 replications^26^.

### Fractionation of bacterial cells

Outer membrane proteins of *V. parahaemolyticus* were obtained as previously described^27^. Briefly, exponential phase cultures (4 ml) were pelleted, lysed by sonication and then centrifuged at 20,000 g for 2 min. Supernatants were then transferred to fresh tubes and centrifuged again (20,000 g, 30 min, 4 °C). The pellets were re-suspended in 500 μl 1% sodium lauryl sulfate in 10mM HEPES (pH7.4) and incubated at room temperature for 30 min. Outer membrane proteins were obtained after centrifugation at 20,000 g for 30 min (4 °C). The detergent soluble and insoluble fractions of the outer membrane proteins were separated by SDS-PAGE on 11% polyacrylamide gel, transferred to PVDF membrane, and subjected to Western Blotting using rabbit α-VpadV polyclonal antibody (1:2000, Pierce).

### Expression and purification of recombinant proteins

Different fragments of VpadF were amplified by PCR using the primers listed in **ST 2** and cloned into pET28 vector. Recombinant proteins were induced by IPTG and then purified using affinity chromatograph methods as previously described^28^, dialyzed into PBS buffer and examined by SDS–PAGE. Protein concentrations were measured by comparison with the BSA standards (Amresco).

### Solid phase binding assay

One hundred μl each of recombinant fibrinogen (F3879, sigma), full-length fibronectin (F2006, Sigma), HBD (F9911, Sigma) and CBD (F0162, 45-kDa, Sigma) domains of fibronectin at a concentration of 5 μg/ml in 50mM Na_2_CO_3_, pH 9.6 was coated onto ELISA plates (IWAKI) at 4 °C overnight. After three-time washes with PBS, the plate was blocked with 1% (w/v) BSA in 50 mM Na_2_CO_3_ (pH 9.6) and incubated at room temperature for 1h. The ELISA plate was then washed three times and then incubated with 3xFlag-tagged, recombinant VpadF truncation fragments at room temperature for 1 h. After triple washes, 100 μl mouse α-3xFlag-HRP antibody (diluted 1:1000 0) was added to each well and incubated at room temperature for 1 h. 100 μl TMB (T4444, sigma) was added to each well for color development (5 min, room temperature). After quenching with 100 μl H_2_SO_4_ (1 M), absorbance was read at 450 nm. BSA was coated simultaneously as negative control.

### Cell attachment and cytotoxicity assays

HeLa and HT-29 cells were maintained in Dulbecco’s Modified Eagle Medium (DMEM, Invitrogen) supplemented with 10% fetal bovine serum (FBS, Invitrogen) at 37 °C in 5% CO2. Attachment assay was carried out as previously described^29^. Briefly,70–90% confluence cells were infected with freshly prepared *V. parahaemolyticus* at a multiplicity of infection (MOI) of ~10 CFU/cell. To consider the factor that *V. parahaemolyticus* may replicate during the experiment, bacterial cell were added to the empty wells of the cell culture plates and incubate for the same time as the binding experiment to determine the final total number of *V. parahaemolyticus* for the binding experiment. After 1 h infection, cells were washed three times with PBS, lysed with 1% Triton X-100 at 37 °C for 10 min to get the successful attached bacteria. The cell lysates and control bacteria were serially diluted and plated on LBS agar. Attachment rate was calculated by dividing bound bacteria to the total bacterial load.

For cytotoxicity assay, similar conditions were used as mentioned above. The supernatants from infected epithelial cell cultures (50–80% confluence) were collected at specific time points. The amounts of LDH release were determined using CytoTox 96 Non-Radioactive Cytotoxicity kit (Promega) following the manufacturer’s instructions. The LDH activity in the supernatant of uninfected cells was also measured to obtain a spontaneous LDH value. Percent cytotoxicity was calculated with the following formula: (test LDH release-spontaneous release)/(maximal release-spontaneous release).

### RT-PCR

Overnight *V. parahaemolyticus* culture was firstly diluted 1:100 in fresh LBS broth and grown to exponential growth phase (OD600 ≈ 0.5–0.7). Cells were collected and re-suspended in the same volume of DMEM with different concentration of FBS (0, 5% and 10% FBS), respectively. After incubation for 30 min at 37 °C, bacteria were collected. Exponential growth phase culture that grown in LBS was directly collected. RNA was extracted using Trizol (Invitrogen) following the manufacturer’s instructions. Residual DNA was removed from the sample with DNase (Turbo DNase, Ambion). RT-PCR reactions were performed using Superscript one-step RT-PCR system (Invitrogen) following the manufacturer’s instructions. Primer pairs, rtVpadF-F/rtVpadF-R and rtrpoA-F/rtrpoA-R, were used to amplify the target genes, respectively (**ST 2**).

### Murine Infection assay

*V. parahaemolyticus* strains (10^8^ CFU) were intraperitoneally injected into 6- to 10-week-old C57BL/6 mice (n = 10) as described previously^30^,^31^,^32^ and the survival of mice was measured at the indicated time points. Three independent experiments were performed. The animal experiments were conducted in the National Institute for Communicable Disease Control and Prevention, Chinese Center for Disease Control and Prevention (CDC) following the guidelines and policies approved by China CDC.

### Statistical analysis

Statistical analysis was performed using Prism software (version 5.0, GraphPad software). The data were the averages of three independent experiments. A *P* value of 0.05 or lower was considered significant. For analysis of the murine survival ratio, Kaplan-Meier and log rank tests were performed and *P* values of <0.01 was considered statistically significant.

## Results

### *VpadF* is probably a adhesin gene in *V. parahaemolyticus*

To identify potential adhesins, we searched the genome sequence of a pandemic strain RIMD2210633. An open-reading frame, *vp1767*, designated *VpadF*, which encodes a putative protein with 736 amino acid residues, attracted our attention. VpadF consists of a putative transmembrane domain at the N-terminal, followed by five bacterial immunoglobulin-like tandem repeats (Bigs). Sequence alignment analysis showed that Bigs in VpadF possesses ~30% amino acid identity to the last Big of LigA and LigB, which possess 13 and 12 Bigs, respectively (SF3). LigA and LigB were described as adhesive molecules in *Leptospira interrogans*^33^,^34^,^35^,^36^. Bigs are widely present in numerous proteins, especially in surface proteins that involved in pathogen-host cells interactions^36^,^37^. It is likely that VpadF is an adhesive factor.

**Figure 1:**
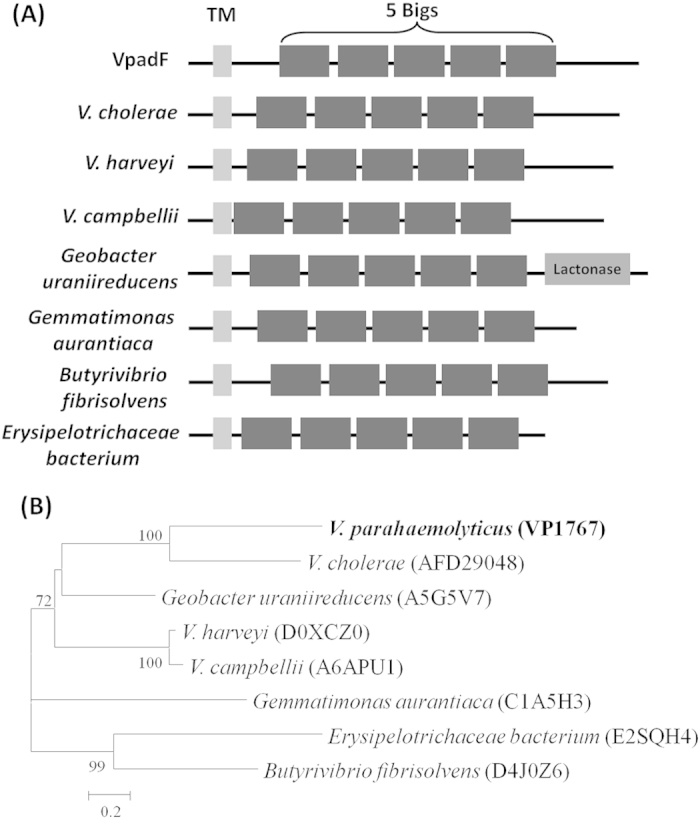
Schematic representation and phylogenetic analysis of *VpadF*. (**A**) Schematic representation of VpadF and its homologues; (**B**) Neighbor-joining tree based on five Bigs sequences showing the phylogenetic relationships of VpadF and its homologues. The protein sequences were obtained from either NCBI or UniProt. Bootstrap values (>50%) are shown at branch nodes. Bar, 0.2 difference at the amino acid level.

Proteins that possess similar domain configuration to that of VpadF also exist in other bacteria, such as *Butyrivibrio fibrisolvens*, *Gemmatimonas aurantiaca*, *V. campbellii*, *V. harveyi*, *etc* (Fig. 1A), suggesting that they share a common ancestor and represent a protein family. Phylogenetic analysis based on the core regions of VpadF and its homologs revealed that VpadF formed a distinct branch related to its homolog from *V. cholerae*, while it was separated from that of *V. harveyi* (Fig. 1B). PCR screening of *VpadF* on environmental and clinical *V. parahaemolyticus* strains showed that almost all isolates possessed this gene with a few exceptional cases (Table 1). BLAST of the 293 available draft genome sequences of *V. parahaemolyticus* in GenBank confirmed that about 256 out of 293 (87%) whole genome sequences contained *VpadF* gene. BLASTN of GenBank non-redundant (NR) database failed to find any high homologues in any bacteria other than *V. parahaemolyticus*. In addition, the codon adaption index (CAI) and expression level analysis of VpadF were found to be normal compared to other genes in *V. parahaemolyticus* (Data not shown). These data suggested that VpadF is likely to be an intrinsic gene in *V. parahaemolyticus* and gets lost in some cases rather than a horizontal gene transferred (HGT) gene.

### VpadF localizes to the outer membrane

**Figure 2:**
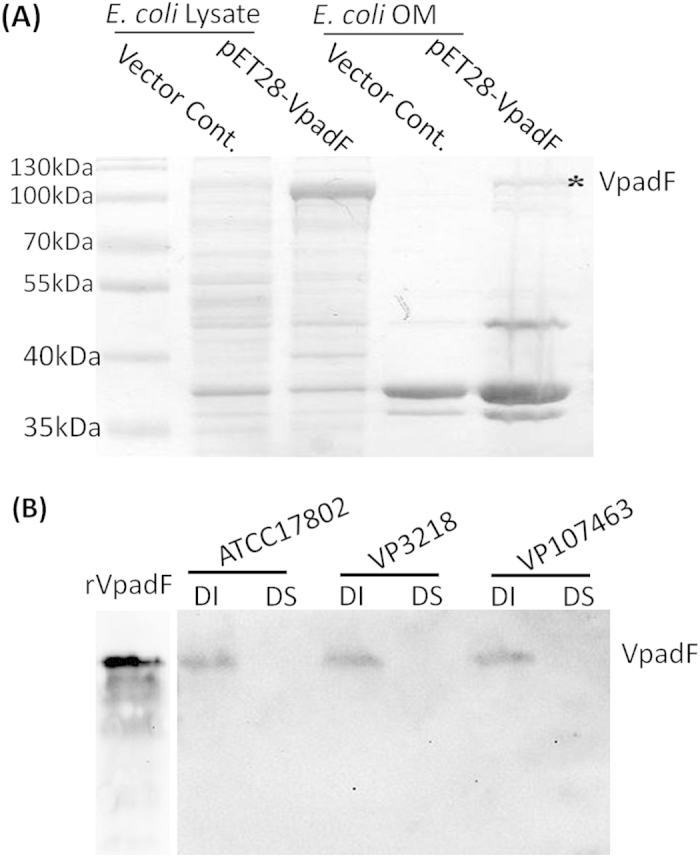
VpadF is an outer membrane protein. (**A**) Fractionation and visualization of VpadF in the outer membrane fraction of *E. coli* BL21 by SDS-PAGE. (**B**) Western blot analysis of the outer membrane fractions of different clinical *V. parahaemolyticus* strains using anti-rabbit polyclonal antibody to VpadF.

Firstly, we tried to find the appropriate condition for endogenous *VpadF* expression because some adhesin genes are not able to be expressed *in vitro*, or just transcribed under specific condition^38^,^39^. As shown in Supplementary Figure 1, the transcription of *VpadF* was stimulated in DMEM media in a FBS-independent manner, while its expression was inhibited in LBS broth. We then analyzed the localization of VpadF by heterogeneous expression in *E. coli* BL21. After Coomassie blue staining of the SDS-PAGE gel, a ~110 KDa protein band was clearly visible from the outer membrane fractions of *E. coli* BL21 expression VpadF (Fig. 2A). To further determine the cellular localization of VpadF in *V. parahaemolyticus*, the outer membrane fractionation of three clinical *V. parahaemolyticus* strains, VPATCC17802, VP3218 and VP1074, were probed with VpadF antibody generated in our lab. It showed that VpadF was localized at the detergent insoluble fraction of the outer membrane of *V. parahaemolyticus* (Fig. 2B).

### VpadF mediates *V. parahaemolyticus* infection to epithelial cells

**Figure 3:**
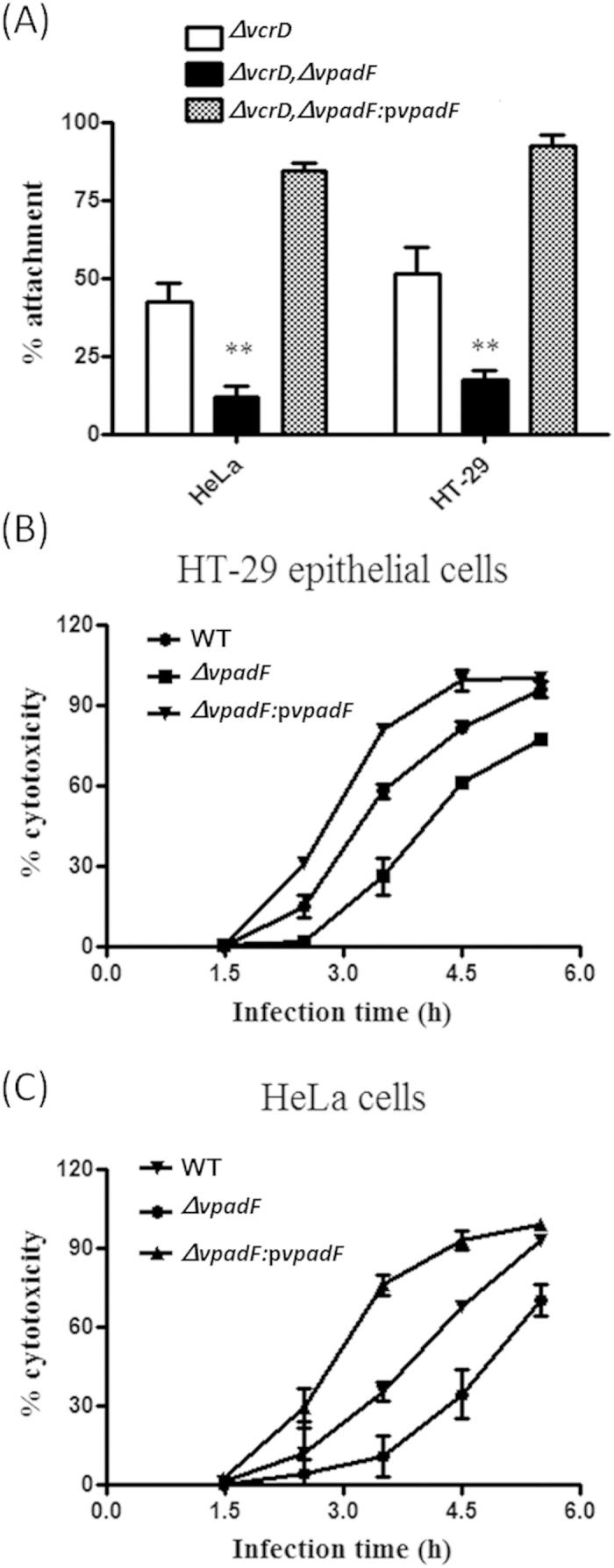
Contribution of VpadF to *V. parahaemolyticus* host cell attachment and cytotoxicity. (**A**) Adhesion of different strains of *V. parahaemolyticus* including wild type (WT), *VpadF* deletion mutant (Δ*VpadF*) and *VpadF* deletion mutant complementary strain (Δ*VpadF*:*pVpadF*) to HeLa and HT-29 epithelial cells. Values represent the mean ± the SE of three independent experiments. Statistical comparisons were performed with a one-way analysis of variance (ANOVA) with Tukey’s multiple comparison tests. **p < 0.01. Cytotoxicity of different strains of *V. parahaemolyticus* including wild type (WT), *VpadF* deletion mutant (Δ*VpadF*) and *VpadF* deletion mutant complementary strain (Δ*VpadF*:*pVpadF*) to HeLa (**B**) and HT-29 epithelial cells (**C**). The LDH released from lysed HeLa and HT-29 epithelial cells were measured at specific time points. Values represent the mean + the SE of three independent experiments.

Having confirmed that VpadF is a surface protein, we then assessed the role of VpadF on *V. parahaemolyticus* attachment to HeLa and HT-29 epithelial cells. A Δ*vcrD1* background strain (T3SS1 negative), which showed a much slower cell lysis rate, was constructed as wild type (WT) *V. parahaemolyticus* caused cell lysis quickly after infection, making it unfeasibl for the attachment assay^40^. The deletion of *VpadF* in the Δ*vcrD1* strain dramatically decreased its attachment to HeLa and HT-29 epithelial cells (Fig. 3B). The complementary strain, Δ*VpadF*Δ*vcrD1*:p*VpadF*, showed ~3 fold higher cell adherence ratio than that of the Δ*VpadF*Δ*vcrD1* strain (Fig. 3B), suggesting that VpadF is required for bacterial cell attachment and *in trans* expression of *VpadF* increases its cell attachment ratio.

To test whether the host cell adherence mediated by *VpadF* affects cytotoxicity of *V. parahaemolyticus* against the epithelium, the lysis of HeLa and HT-29 epithelial cells were measured by monitoring the release of lactate dehydrogenase (LDH) after infection with WT *V. parahaemolyticus*, Δ*VpadF* strain (*vcrD1* positive) and the complementary strain Δ*VpadF*:p*VpadF*. At 3.5 h, Δ*VpadF* caused a ~50% decreased cytotoxicity compared to that caused by WT and Δ*VpadF*::*pVpadF*. After 5.5 h infection, HeLa and HT-29 epithelial cells were nearly completely lysed by WT and the VpadF complementary strains, while Δ*VpadF* infected cells showed ~30% less lysis (Fig. 3C). This suggested that VpadF plays an important role in epithelial cell infection.

### VpadF is responsible for lethality in mice

**Figure 4:**
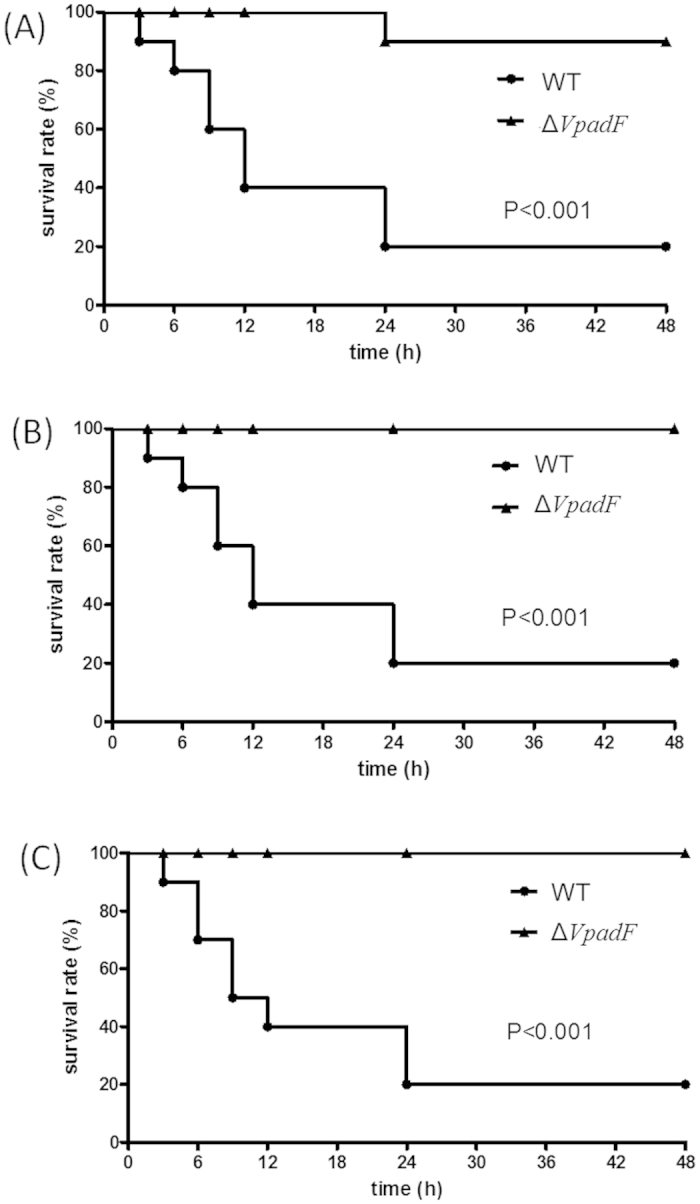
Survival rates of murine models infected with *V. parahaemolyticus* strains. C57BL/6 mice (n = 10) were infected intraperitoneally with WT or Δ*VpadF* strains (10^8^ CFU) and the dead mice were recorded at specific time points during a 96 period of time. Only the data for the first 48 h was shown since no further change was observed in these mice for the next 48 h. Data from three independent experiments, (**A**–**C**), were shown. Kaplan-Meier and log rank tests were used to analyze the data (P < 0.001).

To explore whether VpadF affects *V. parahaemolyticus* pathogenesis, mice were infected with WT *V. parahaemolyticus* VP3218 and its *VpadF* deletion mutant, respectively. As shown in Fig. 4A–C, mice infected with WT strain displayed only 20% survival, while deletion of *VpadF* nearly completely abolished the lethality in mice after 48 h infection. After 96 h infection, no further lethal effect was observed in the mice infected by WT *V. parahaemolyticus* VP3218 or its *VpadF* deletion mutant (data not shown). This indicated that VpadF mediating *V. parahaemolyticus*-host interaction is required for its pathogenesis in mice.

### VpadF binds both fibronectin (Fn) and fibrinogen (Fg)

**Figure 5:**
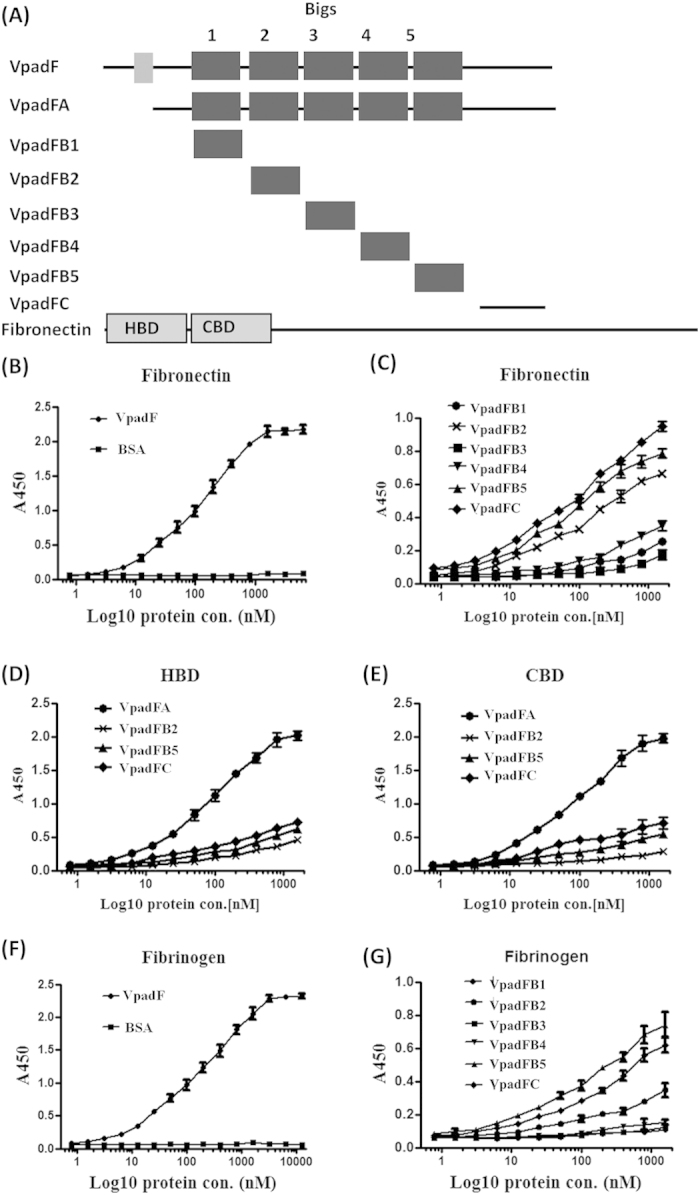
Characterization of interactions between VpadF and its cell surface receptors. (**A**) Schematic representation of VpadF, its truncation domains and fibronectin; VpadFA (residues 37–736), VpadFB1 (residues 98–174), VpadFB2 (residues 184–255), VpadFB3 (residues 272–348), VpadFB4 (residues 356–433), VpadFB5 (residues 442–522) and VpadFC (residues 625–736); (**B**) Binding of rVpadFA to fibronectin; (**C**) Binding of various domains of rVpadF, rVpadFB1, B2, B3, B4, B5 and C to fibronectin; (**D,E**) Binding of rVpadFA, B2, B5 and C to HBD and CBD of fibronectin; (**F**) Binding of rVpadFA to fibrinogen; (**G**) Binding of various domains of rVpadF, rVpadFB1, B2, B3, B4, B5 and C to fibrinogen. BSA was used as negative control. Values represent the mean + the SE of three independent experiments.

To gain further insight into the mechanism by which VpadF mediates host cells attachment and thus contributes to the pathogenesis, we set out to identify potential host receptors of VpadF. It was suggested that besides *mam7*, *V. parahaemolyticus* produces other adhesins to bind Fn^29^. Initially, we assessed the Fn binding ability of VpadF. VpadFA, the VpadF truncated fragment that lack of 36 N-terminal amino acid residues was first purified (Fig. 5A and SF2). The specific interaction between VpadFA and Fn was examined using the solid-phase binding assay. As shown in Fig. 5B, VpadFA was able to interact with Fn in a pronounced dose-dependent manner and reached a saturated status, with a Kd (the concentration able to saturate 50% of substrate) of ~200 nM. To evaluate which region in VpadF contributes to its binding property, all five Big repeats, VpadFB1 (the first Big domain), VpadFB2 (the second Big domain), VpadFB3 (the third Big domain), VpadFB4 (the fourth Big domain), VpadFB5 (the fifth Big domain) and the C-terminal end, VpadFC, were individually purified, respectively. Our results demonstrated that VpadFB2, VpadFB5 and VpadFC strongly bound to Fn and VpadFC displayed highest binding affinity. In contrast, VpadFB1, VpadFB3 and VpadFB4 showed weak binding activities to Fn (Fig. 5C).

To further demonstrate which parts of Fn are bound by VpadF, we tested the binding activities of VpadFA, B2, B5 and C to the immobilized N- terminal 30-KDa (heparin binding domain, HBD) and 45-KDa fragments (gelatin and collagen binding domain, CBD) of Fn. As shown in Fig. 5D,E, both HBD and CBD were targeted by VpadFA, B2, B5 and C. VpadFB5 and VpadFC bound HBD and CBD with similar avidities, while higher than those of VpadFB2.

Septicemia caused by *V. parahaemolyticus* is potential fatal to immunocompromised and liver failure individuals^41^. In Gram-positive pathogens, septicemia is related to their binding abilities to Fg since Fg is not only rich in human blood but also present in extracellular matrix^42^,^43^. Thus, we tested the binding ability of VpadF to Fg. Our results displayed that Fg was bound by VpadFA, with a Kd of ~400 nM (Fig. 5F). Similar to VpadF binding Fn, this protein also interacted with Fg mainly dependent on its C-terminal region, VpadFB5 and VpadFC (Fig. 5G).

## Discussion

Initial host cell adhesion is the first important step in bacterial pathogenesis. In the case of *V. parahaemolyticus*, its infectious processes require the adherence to intestinal epithelial cells, resulting in epithelial cell extrusion, villus disintegration and formation *V. parahaemolyticus*-filled cavities^44^. Adhesins play a pivotal role in the tight bacteria-host cells attachment to initiate the downstream processes. In the present study, we discover a novel adhesin gene, *VpadF*, that plays a significant role on the pathogenesis of *V. parahaemolyticus*.

VpadF has several features that distinguish it from other previously described adhesive factors, such as MSHA pilus, enolase, capsular polysaccharide, T6SSs and MAM7 in *V. parahaemolyticus*. Firstly, this protein shows a unique domain organization. Secondly, VpadF is required for the lethal effects of *V. parahaemolyticus* on mice. Moreover, VpadF is a multifunctional adhesin that is capable of interact with both Fn and Fg. To the best of our knowledge, VpadF is the first reported adhesin that be able to bind Fg in *Vibrio* species. It was shown that Fg binding proteins have essential roles in pathogenesis by enabling bacteria to penetrate host barriers and spread in tissues^43^. VpadF is likely to both strengthen the attachment of *V. parahaemolyticus* to epithelial cells and modulate its spreading in the infected tissues. The molecular mechanism of the interactions between VpadF and host receptors is also different from those of other adhesins in *Vibrio*. For instance, OmpU from *V. vulnificus* recognizes RGD tripeptide that localizes the middle part of Fn and MAM7 interact with HBD of Fn^29^,^45^. In contrast, VpadF can bind the whole N-terminal fragments, HBD and CBD of Fn.

VpadF also differs from Bigs possessing adhesins in *Leptospira interrogans*. Biochemical analysis revealed that the fifth Big repeat in VpadF significantly contributes to Fn and Fg binding, while in LigA and LigB, their binding avidities are mainly dependent on the 13^th^ and 12^th^ Big domain, respectively^46^,^47^,^48^. Moreover, unlike in LigA and LigB, only the last Bigs can recognize host components, the 2^nd^ Big repeat in VpadF also exhibited Fn/Fg binding property to some extent. These results implicates the number of Bigs is not the primary determinant of function and individual Big repeat in a single protein could be functional diverged even each Big fold shares some sequence similarity with each other (SF3).

In conclusion, we identified and characterized a novel and essential adhesion factor from *V. parahaemolyticus* and demonstrated its significant role on host cell attachment, cytotoxicity and pathogenicity. VpadF is a potential good candidate for *V. parahaemolyticus* infection control for both vaccine development and drug target.

## Additional Information

**How to cite this article**: Liu, M. and Chen, S. A novel adhesive factor contributing to the virulence of *Vibrio parahaemolyticus*. *Sci. Rep.* **5**, 14449; doi: 10.1038/srep14449 (2015).

## Supplementary Material

Supplementary Information

## Figures and Tables

**Table 1:** Distribution of the *VpadF* gene among *V. parahaemolyticus* strains.

**Genotype**	**Total No.**	**No. positive**	**Prevalence, %**
Clinical strains (*tdh*^+^)	34	28	82
Environmental strains (*tdh*^−^, *trh*^−^)	33	33	100
