# Supplementary material for: A novel adhesive factor contributing to the virulence of Vibrio parahaemolyticus
Source: Sci Rep. 2015 Sep 24;5:14449. doi: 10.1038/srep14449 (PMC4585867; doi:10.1038/srep14449)
Supplement: Supplementary Information [file srep14449-s1.doc]

**Supplementary Appendix**

**A novel adhesive factor contributing to the virulence of *Vibrioparahaemolyticus***

Ming Liu, Sheng Chen*

^1^ Shenzhen Key Lab for Food Biological Safety Control, Food Safety and Technology Research Center, Hong Kong PolyU Shenzhen Research Institute, Shenzhen, China;

^2^State Key Laboratory of Chirosciences, Department of Applied Biology and Chemical Technology, The Hong Kong Polytechnic University, Hong Kong, China;

*Correspondence: Sheng Chen, Department of Applied Biology and Chemical Technology, The Hong Kong Polytechnic University, Hung Hom. Kowloon, Hong Kong, China; Email: [sheng.chen@polyu.edu.hk](mailto:sheng.chen@polyu.edu.hk)

**Supplementary Table 1 (ST1). Bacteria and plasmids used in this study**

| Strain or plasmid | Description | Reference or source |
| --- | --- | --- |
| *E.coli* |  |  |
| SY327 λ*pir* | Δ(*lac-pro*) *argE*(Am) *rifmalA recA56*λ*pir* | [^51^](#_ENREF_51) |
| DH5α | *recA1 endA1 gyrA96 thi1 hsdR17 supE44 relA1 lacZΔM15* | Lab collection  Lab collection |
| BL21 (DE3) | F^−^*ompThsdS gal* | Lab collection |
| *V. parahaemolyticus* |  |  |
| VP3218 | clinical isolate, *tdh*^+^, *t3ss1*^+^ , *t3ss2*^+^ | [^14^](#_ENREF_14) |
| Δ*VpadF* | *vp1767* gene deletion mutant | This study |
| Δ*vcrD1* | *vp1662* gene deletion mutant | This study |
| Δ*vcrD1*Δ*VpadF* | *vp1662* and *vp1767* genes deletion mutant | This study |
| Δ*vcrD1*Δ*VpadF*:p*VpadF* | Δ*vcrD1*Δ*VpadF* complemented with *vp1767*gene | This study |
| Δ*VpadF*:p*VpadF* | Δ*VpadF* complemented with *vp1767*gene | This study |
| Plasmids |  |  |
| pDM4 | Cm^r^; suicide vector with an R6K origin and *sacBR* genes from *Bacillus subtilis* | [^52^](#_ENREF_52) |
| pMMB207 | RSF1010 derivative, *IncQlacI*^q^Cm^r^P*tacoriT* | [^52^](#_ENREF_52) |
| pPK2013 | Km^r^Tra^+^ Mob^+^, ColE1 replicon | [^53^](#_ENREF_53) |
| pET28D3 | Km^r^, 6xHistidine tag, 3xFlag tag | Lab collection |

**Supplementary Table 2(ST2). Primers used in this study.**

| Primers | DNA sequence or reference | |
| --- | --- | --- |
| Knock out *VpadF* | | |
| vp1767-1F | | CCGCTCGAGCGAATTGAGCACTTCCCATT |
| vp1767-1R | | TTACTTTCACTAACTTTCAATCAAACTTTTATTATTAGAC |
| vp1767-2F | | GTCTAATAATAAAAGTTTGATTGAAAGTTAGTGAAAGTAA |
| vp1767-2R  Knock out *vcrD1* | | GCTCTAGAAACTGCCTGAGCTCGTTGTT  [^54^](#_ENREF_54) |
| Complement *VpadF* | | |
| vp1767com-F | CGAGCTCTAAGGAGGTAGGATAATAATGTTTGACTCTATGATA | |
| vp1767com-R | CGGGATCCTTACTTGTCATCGTCATCCTTGTAATCCTTAAGAGGAACGCCAG | |
| Expression | | |
| VpadF-F | CGAGCTCATGCCACCAATGAGCTTGCCAG | |
| VpadF-R | CGGGATCCTTACTTAAGAGGAACGCCA | |
| VpadFB1-R | CGGGATCCTTATTCGCTACTGAACTCATCT | |
| VpadFB1-F | CGAGCTCATGGTGTCTCGTATTGCGCTA | |
| VpadFB2-R | CGGGATCCTTAATCAAGCTCGGCATAGATAT | |
| VpadFB2-F | CGAGCTCATGTTGACCAACATTTACTTCG | |
| VpadFB3-R | CGGGATCCTTATTGTATTGGCTCGCTTTGC | |
| VpadFB3-F | CGAGCTCATGGTGAGTATGGACATTTCGT | |
| VpadFB4-R | CGGGATCCTTACAATGGTGCACTGGTGATCG | |
| VpadFB4-F | CGAGCTCATGCTAACAAATATTGAAGTGA | |
| VpadFB5-R | CGGGATCCTTACTTGACCTCAGAGGTGCCT | |
| VpadFB5-F | CGAGCTCATGTTAGAAAAAATCCATGTTC | |
| VpadFC-F | CGAGCTCATGGCAAACTGGTGTCATGATT | |
| VpadF-F | CGAGCTCATGTTTGACTCTATGATAA | |
| RT-PCR | | |
| rthadV-F | TCCTCACGAGTTTGTTTGGT | |
| rthadV-R | GGAATAACGGGATCAGAAGGC | |
| rtrpoA-F | TCGCCGCATTCTTCTATCTT | |
| rtrpoA-R | TCAGCGTTGTCATCCGTTAG | |

**
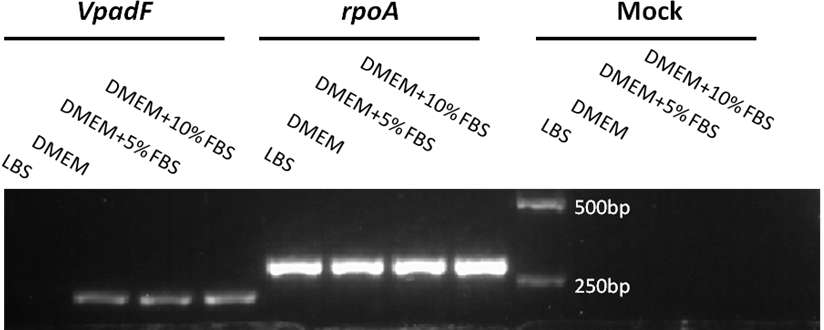
**

**Supplementary Figure 1 (SF1).Detection of *VpadF* expression by RT-PCR in *V. parahaemolyticus*.**RT-PCR showing expression of *VpadF*.*RpoA* was a house-keeping gene used as an internal control to ensure that RNA was present in all samples. Mock reactions, which did not contain reverse transcriptase, were used as control against genomic contamination of the RNA preparations.

**
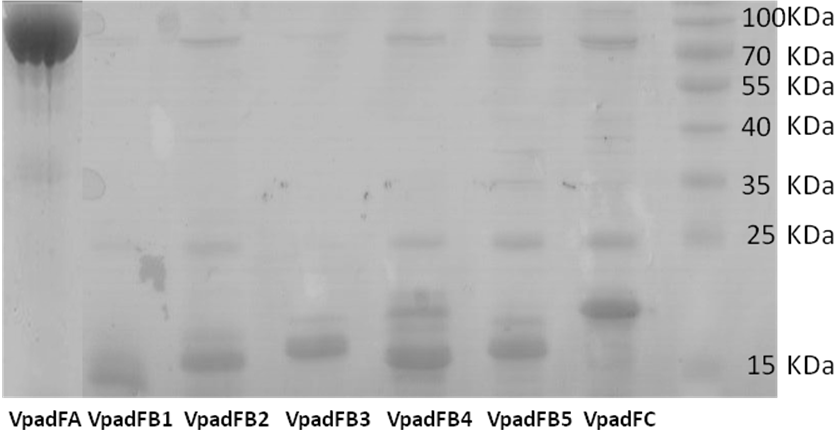
**

**Supplemental Figure 2 (SF2).SDS-PAGE of purified recombinant proteins of VpadF.**

**
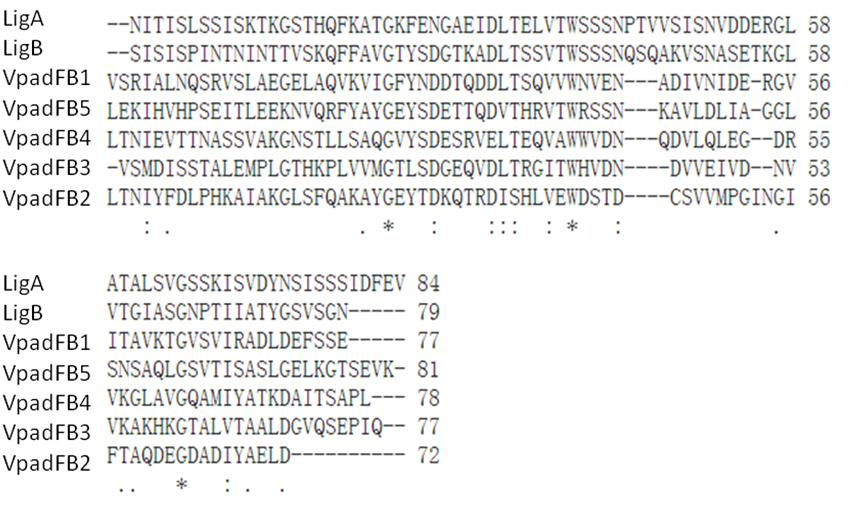
**

**Supplemental Figure 3 (SF3).Multiple sequence alignment of Big repeats.** Individual Big domain in VpadF and the last Big folds in LigA and LigB (GenBank accession No. ACH89909 and ACH89908, respectively) were aligned using the CLUSTAL W2.
